# Supplementary material for: The Batten disease gene product CLN6 is a glycerophosphoglycerol epimerase that establishes S,S-BMP stereochemistry
Source: bioRxiv. 2026 Oct 9:2026.10.07.757477. Preprint. [Version 1] doi: 10.64898/2026.10.07.757477 (PMC13692256; doi:10.64898/2026.10.07.757477)
Supplement: Supplementary file 1 [file NIHPP2026.10.07.757477v1-supplement-4.pdf]

bioRxiv preprint doi: https://doi.org/10.64898/2026.10.07.757477; this version posted October 9, 2026. The copyright holder for this preprint (which was not certified by peer review) is the author/funder, who has granted bioRxiv a license to display the preprint in perpetuity. It is made available under a CC-BY 4.0 International license.

Supplementary Figures

Figure S1 Validation of CLN6-deficient cell models.

(A) Immunoblot of CLN6 and vinculin in WT and *CLN6*-KO HEK293T cells.

(B) Immunoblot of CLN6, Twin-Strep-tagged CLN6 (detected with anti-Strep antibody) and vinculin in WT, *CLN6* KO and *CLN6*-KO HEK293T cells expressing WT CLN6 or the patient R62H and H127Y variants, C-terminus of each is fused to Twin-Strep tag.

(C) Representative immunofluorescence images of *CLN6*-KO HEK293T cells expressing Strep-tagged WT CLN6, R62H, or H127Y. Strep-tagged CLN6 is shown in green, the ER marker calnexin in magenta, and nuclei stained with DAPI in blue. Merged images are shown on the right. Scale bars, 20 µm.

(D) Quantification of Manders' colocalization coefficient between CLN6-2XStrep and calnexin from **(C)**. Each dot represents one imaging field. Data are presented as mean ± SD, *n* = 4 fields for WT, 5 fields for R62H, and 3 fields for H127Y.

(E) Immunoblot of CLN6 and vinculin in WT and *CLN6* knockout iPSCs.

(F) Targeted analysis of BMP species in WT iPSCs and two independent *CLN6*-KO clones (cl1 and cl2). Data are presented as mean ± SD; *n* = 5 biologically independent samples for the KO clone 2 and *n* = 4 for each remaining group.

(G) Targeted lipidomics analysis of LPG species in WT HEK293T cells, *CLN6*-KO cells, and KO cells re-expressing WT CLN6 or patient-derived variants, R62H or H127Y. Data are presented as mean ± SD; *n* = 3 biologically independent samples for the KO group and *n* = 4 for each remaining group.

(H and I) Targeted analysis of LPG species in WT cells and two independent *CLN6*-KO clones (cl1 and cl2), measured in iPSCs **(H)** and iNeurons **(I)**. Data are presented as mean ± SD; *n* = 5 biologically independent samples for iPSC KO clone 2 and *n* = 4 for each remaining group.

In **(F–I)**, the abundance of each lipid species was first normalized to endogenous POPC and then normalized to its mean abundance in the corresponding WT group. Statistical significance in **(F–I)** was assessed using two-way ANOVA; **P* < 0.05, ***P* < 0.01, and *****P* < 0.0001.

bioRxiv preprint doi: https://doi.org/10.64898/2026.10.07.757477; this version posted October 9, 2026. The copyright holder for this preprint (which was not certified by peer review) is the author/funder, who has granted bioRxiv a license to display the preprint in perpetuity. It is made available under a CC-BY 4.0 International license.

Figure S2 Development of chiral chromatographic methods to resolve the stereochemistry and regiochemistry of BMP and LPG.

(A) Representative extracted ion chromatograms (EICs) of derivatized synthetic BMP 18:1_18:1 standards: *R*,*R*-2,2′ -BMP, *R*,*S*-2,2′ -BMP, *S*,*S*-2,2′ -BMP, and *S*,*S*-3,3′ -BMP, shown in blue, beige, yellow, and magenta, respectively. Impurities are found in some standards because of common acyl migration.

(B) Representative EICs of derivatized synthetic deuterium-labeled LPG 18:1 standards: d5-*R*,*S*-2-LPG, d5-*R*,*S*-1-LPG, d5-*S*,*R*-2-LPG, d5-*S*,*S*-2-LPG, and d5-*S*,*S*-3-LPG, shown in orange, magenta, blue, beige, and yellow, respectively. Impurities are found in some standards because of common acyl migration.

Each trace is independently normalized to its maximum peak intensity (100%). Dashed lines indicate the retention times of the assigned isomers. Traces are vertically offset for clarity.

Figure S3 Method for GPG stereochemistry, validation of CLN8-deficient cells, and BMP responses to *S*,*S*-GPG supplementation.

(A) Workflow for analyzing GPG stereochemistry by chemical derivatization and nanoLC–ion mobility–mass spectrometry using a timsTOF HT instrument.

(B) CRISPR-Cas9 mediated deletion of CLN8 in single and combinatorial knockout cell lines. Alignment of the wild-type (WT) *CLN8* reference sequence with edited sequences in *CLN8* knockout and *CLN6/8* double-knockout cells (top), and *CLN5/8* double-knockout and *CLN5/6/8* triple-knockout cells (bottom). The edited alleles contain 219-bp and 292-bp disrupted nucleotides, respectively. gRNA target sequences are highlighted in red, with corresponding gRNAs indicated above. Flanking sequences retained in the edited alleles are shown in black. Ellipses indicated omitted sequence. *CLN5*/8 double-knockout was generated on a previously reported *CLN5* knockout (*21*), and *CLN5*/6/8 triple-knockout was generated based on the *CLN5*/8 double-knockout. *CLN6*/8 double-knockout was generated based on the *CLN8* knockout.

(C) Targeted analysis of BMP species in WT and *CLN8*-knockout (KO) cells. The abundance of each BMP species was first normalized to endogenous POPC and then normalized to its mean abundance in the WT group. Data are presented as mean ± SD of $n = 6$ biologically independent samples. Statistical significance was assessed using two-way ANOVA. ****$P < 0.0001$.

(D) Concentration-dependent changes in indicated BMP abundances following supplementation with exogenous *S*,*S*-GPG in WT iNeurons and iNeurons derived from two independent *CLN6*-KO clones (cl1 and cl2). Abundances are expressed as fold changes relative to the corresponding untreated group (0 µM). Black, pink, and purple curves represent WT, KO clone 1, and KO clone 2, respectively. Data are presented as mean ± SD. $n = 3$ independent biological samples.

bioRxiv preprint doi: https://doi.org/10.64898/2026.10.07.757477; this version posted October 9, 2026. The copyright holder for this preprint (which was not certified by peer review) is the author/funder, who has granted bioRxiv a license to display the preprint in perpetuity. It is made available under a CC-BY 4.0 International license.

(E) Immunoblot validation of CLN6 loss in two independent *CLN6*-knockout ARPE-19 clones (cl1 and cl2), with WT cells included as a control. Vinculin serves as a loading control.

(F and G) Representative fluorescence images of vehicle-treated WT ARPE-19 cells and two independent *CLN6*-knockout clones (*CLN6* KO_cl1 and *CLN6* KO_cl2) treated with vehicle (H_2O) or 400 µM *S,S*-GPG for 10 days. Cells were stained with filipin to visualize cholesterol **(F)** or a mouse anti-GM2 antibody to detect ganglioside GM2 **(G)**. Scale bars, 20 µm.

(H and I) Quantification of filipin and GM2 fluorescence from **(F)** and **(G)**. Fluorescence intensity was normalized to cell area and expressed relative to the mean of vehicle-treated WT cells. Each dot represents one cell, and n denotes the total number of cells analyzed from 4-5 randomly selected imaging fields per condition. For filipin, $n = 39$ for H_2O treated WT cells, 68 for H_2O treated *CLN6* KO_cl1, 89 for *S,S*-GPG *CLN6* KO_cl1, 60 for H_2O treated *CLN6* KO_cl2, and 31 for *S,S*-GPG treated *CLN6* KO_cl2. For GM2, $n = 41$ for H_2O treated WT cells, 64 for H_2O treated *CLN6* KO_cl1, 100 for *S,S*-GPG *CLN6* KO_cl1, 45 for H_2O treated *CLN6* KO_cl2, and 27 for *S,S*-GPG treated *CLN6* KO_cl2. Statistical significance was assessed using one-way ANOVA; $^{**}P < 0.01$ and $^{****}P < 0.0001$.

Panel A was created using BioRender (https://biorender.com).

Figure S4 Purification of CLN6 and functional characterization of variants targeting putative Ca^{2+}- and GPG-binding residues.

(A and B) Size-exclusion chromatography profile of WT CLN6-2×Strep purified from Expi293 cells **(A)** and Coomassie-stained SDS–PAGE analysis of the indicated fractions **(B)**. Numbers above the chromatogram indicate collected fractions. Fractions 5-7 were pooled.

(C and D) Size-exclusion chromatography profile of the H127Y variant CLN6-2×Strep purified from Expi293 cells **(C)** and Coomassie-stained SDS–PAGE analysis of the indicated fractions **(D)**. Fractions 6-10 were pooled.

(E) Immunoblot analysis of purified WT CLN6 and H127Y using an anti-CLN6 antibody. 100 ng of protein was loaded.

(F) Immunoblot validation of WT CLN6 and the indicated variants re-expressed in *CLN6*-KO cells, using anti-CLN6 and anti-Strep antibodies. WT and KO cells are included as controls. Vinculin serves as a loading control. Mut1, D173A/E174A/H178A; Mut2, D173Q/E174Q/H178A; Mut3, Y182A/Y223A.

(G) Representative immunofluorescence images of *CLN6*-knockout (KO) cells expressing the indicated Strep-tagged CLN6 variants. Strep-tagged CLN6 is shown in green, the ER marker calnexin in magenta, and nuclei stained with DAPI in blue. Merged images are shown on the right. Scale bars, 20 µm.

bioRxiv preprint doi: https://doi.org/10.64898/2026.10.07.757477; this version posted October 9, 2026. The copyright holder for this preprint (which was not certified by peer review) is the author/funder, who has granted bioRxiv a license to display the preprint in perpetuity. It is made available under a CC-BY 4.0 International license.

(H) Quantification using Manders' colocalization coefficient of CLN6-2×Strep and calnexin overlap in Mut 1, Mut2, and Mut3 in **(G)**. Each dot represents one imaging field; n = 3 fields for Mut1 and Mut2, n = 4 fields for Mut3.

(I) Targeted analysis of BMP species in WT cells, *CLN6*-KO cells, and KO cells re-expressing WT CLN6 or the indicated variants. The abundance of each BMP species was first normalized to endogenous POPC and then normalized to its mean abundance in the WT group. Data are presented as mean ± SD. n = 4 independent biological samples. Statistical significance was assessed using two-way ANOVA. Asterisks indicate statistical significance: *P < 0.05, ***P < 0.001, and ****P < 0.0001.

Figure S5 Quantitative analysis of BMP and LPG in mouse tissues and clinical samples associated with CLN6 deficiency.

(A–D) Targeted analysis of BMP **(A and B)** and LPG **(C and D)** species in brain **(A and C)** and lung **(B and D)** tissues from heterozygous carrier (HET; *Cln6* WT/*nclf*) and homozygous mutant mice (HOM; *Cln6 nclf/nclf*). The abundance of each lipid species was first normalized to endogenous POPC and then normalized to its mean abundance in the corresponding HET tissue group. Gray and pink bars represent HET and HOM mice, respectively. Data are presented as mean ± SD. n = 5 independent biological samples. Asterisks indicate statistical significance: *P < 0.05, **P < 0.01, ***P < 0.001, and ****P < 0.0001.

(E and G) Relative abundances of BMP **(E)** and LPG **(G)** species in dried blood spots (DBS) from two patients with CLN6 disease in Family One and four healthy controls.

(F and H) Relative abundances of BMP **(F)** and LPG **(H)** species in DBS from two heterozygous carrier parents and their two affected children in Family Two.

(I and J) Relative abundances of LPG **(I)** and BMP **(J)** species in peripheral blood mononuclear cells (PBMCs) from the family shown in **(F and H)**.

In **(E–J)**, the abundance of each lipid species was first normalized to endogenous POPC and then normalized to its mean abundance in the corresponding healthy control group **(E and G)** or carrier-parent group **(F, H, I, and J)**. Gray bars represent healthy controls or carrier parents, and pink bars represent patients or affected children.

Supplementary Tables

Table S1: Untargeted lipidomic analysis of wild-type and *CLN6* knockout HEK293T cells. Related to **Fig. 1A**.

Table S2: MRM transitions for targeted analysis of unlabeled BMP, LPG, and PG.

Table S3: MRM transitions for targeted analysis of derivatized BMP, LPG, and PG.

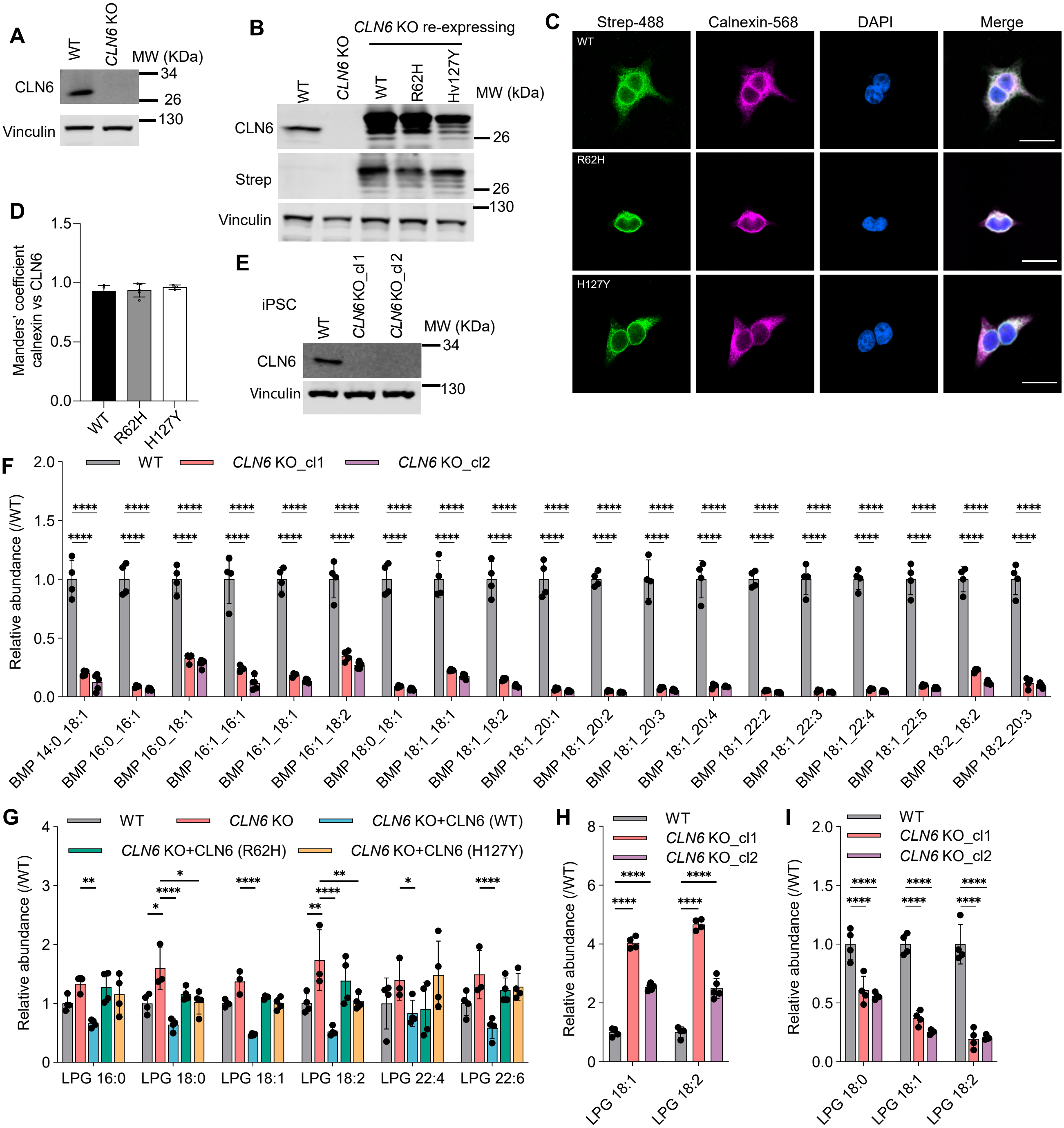
A
WT
CLN6 KO
MW (KDa)
CLN6
Vinculin
34
26
130
B
CLN6 KO re-expressing
WT
CLN6 KO
WT
R62H
Hv127Y
MW (kDa)
CLN6
Strep
Vinculin
26
26
130
C
Strep-488
Calnexin-568
DAPI
Merge
WT
R62H
H127Y
D
Manders' coefficient calnexin vs CLN6
1.5
1.0
0.5
0.0
WT
R62H
H127Y
E
iPSC
WT
CLN6 KO_cl1
CLN6 KO_cl2
MW (KDa)
CLN6
Vinculin
34
130
F
WT
CLN6 KO_cl1
CLN6 KO_cl2
Relative abundance (/WT)
2.0
1.5
1.0
0.5
0.0
****
BMP 14:0_18:1
BMP 16:0_16:1
BMP 16:0_18:1
BMP 16:1_16:1
BMP 16:1_18:1
BMP 16:1_18:2
BMP 18:0_18:1
BMP 18:1_18:1
BMP 18:1_18:2
BMP 18:1_20:1
BMP 18:1_20:2
BMP 18:1_20:3
BMP 18:1_20:4
BMP 18:1_22:2
BMP 18:1_22:3
BMP 18:1_22:4
BMP 18:1_22:5
BMP 18:2_18:2
BMP 18:2_20:3
G
WT
CLN6 KO
CLN6 KO+CLN6 (WT)
CLN6 KO+CLN6 (R62H)
CLN6 KO+CLN6 (H127Y)
Relative abundance (/WT)
4
3
2
1
0
**
*
****
**
*
LPG 16:0
LPG 18:0
LPG 18:1
LPG 18:2
LPG 22:4
LPG 22:6
H
WT
CLN6 KO_cl1
CLN6 KO_cl2
Relative abundance (/WT)
8
6
4
2
0
****
LPG 18:1
LPG 18:2
I
WT
CLN6 KO_cl1
CLN6 KO_cl2
Relative abundance (/WT)
2.0
1.5
1.0
0.5
0.0
****
LPG 18:0
LPG 18:1
LPG 18:2

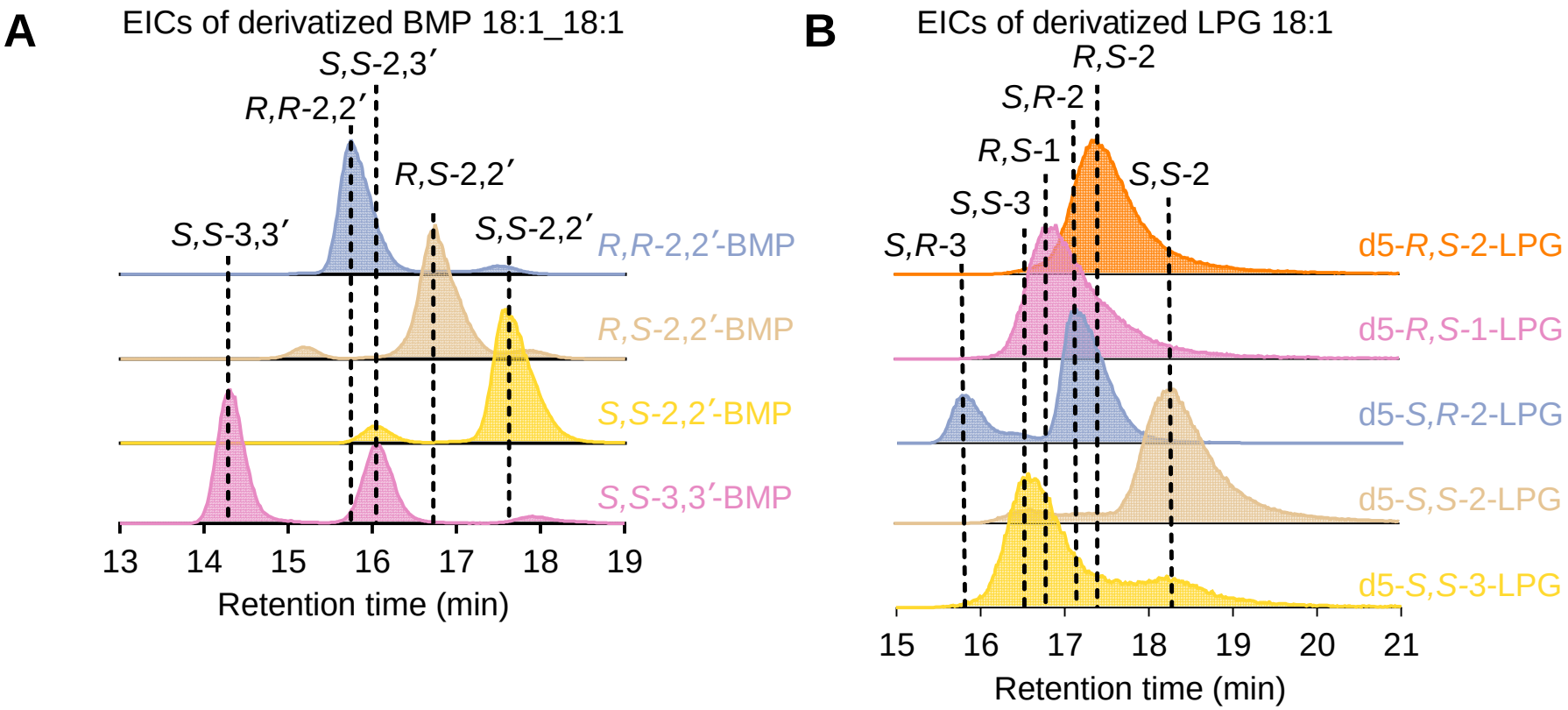
A
EICs of derivatized BMP 18:1_18:1
S,S-2,3′
R,R-2,2′
R,S-2,2′
S,S-3,3′
S,S-2,2′
R,R-2,2′-BMP
R,S-2,2′-BMP
S,S-2,2′-BMP
S,S-3,3′-BMP
13
14
15
16
17
18
19
Retention time (min)
B
EICs of derivatized LPG 18:1
R,S-2
S,R-2
R,S-1
S,S-2
S,S-3
S,R-3
d5-R,S-2-LPG
d5-R,S-1-LPG
d5-S,R-2-LPG
d5-S,S-2-LPG
d5-S,S-3-LPG
15
16
17
18
19
20
21
Retention time (min)

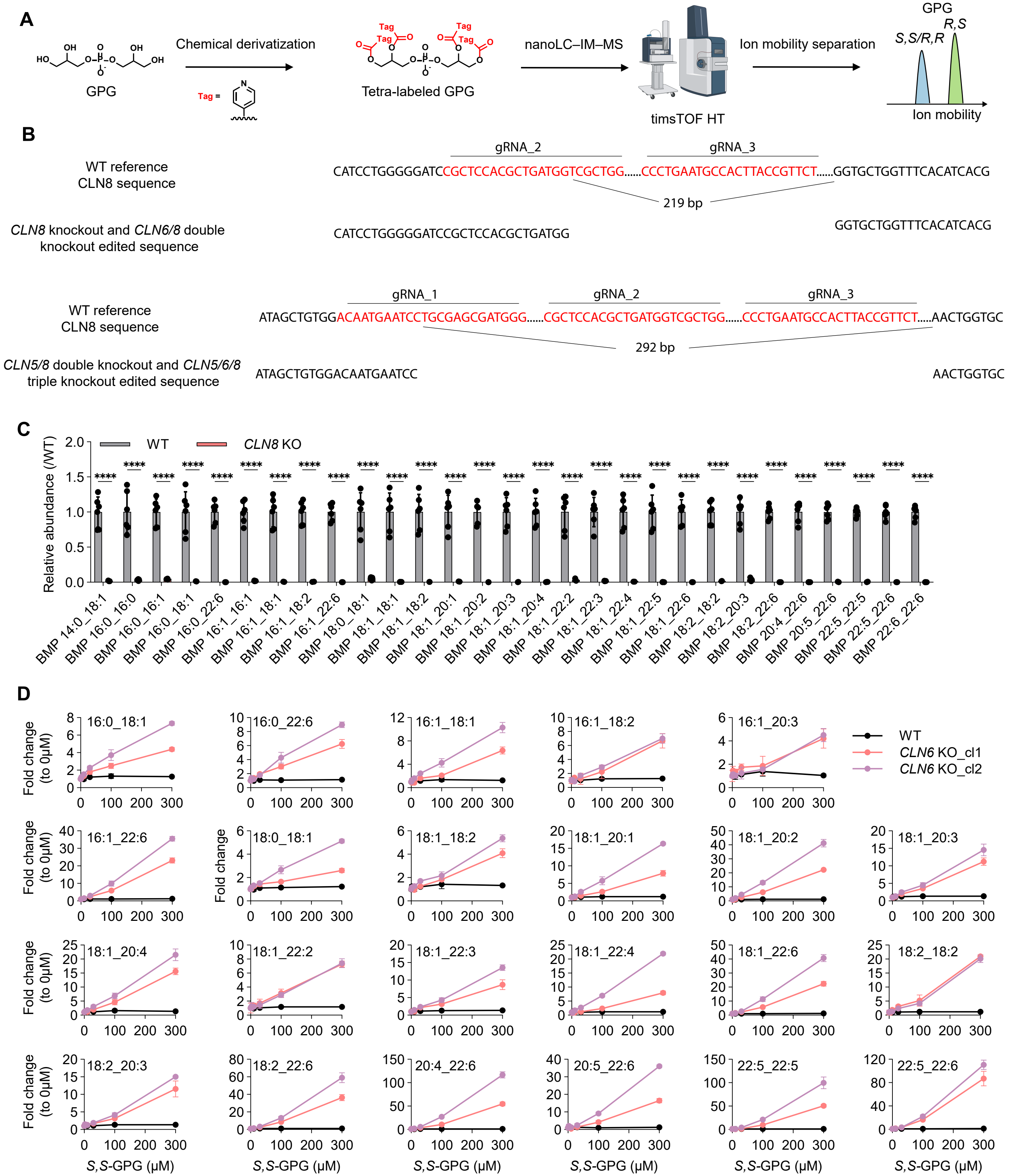
A
Chemical derivatization
GPG
Tag =
Tag
Tetra-labeled GPG
nanoLC–IM–MS
timsTOF HT
Ion mobility separation
GPG
S,S/R,R
R,S
Ion mobility
B
WT reference
CLN8 sequence
gRNA_2
gRNA_3
CATCCTGGGGGATCCGCTCCACGCTGATGGTCGCTGG......CCCTGAATGCCACTTACCGTTCT......GGTGCTGGTTTCACATCACG
219 bp
CLN8 knockout and CLN6/8 double knockout edited sequence
CATCCTGGGGGATCCGCTCCACGCTGATGG
GGTGCTGGTTTCACATCACG
WT reference
CLN8 sequence
gRNA_1
gRNA_2
gRNA_3
ATAGCTGTGGACAATGAATCCTGCGAGCGATGGG......CGCTCCACGCTGATGGTCGCTGG......CCCTGAATGCCACTTACCGTTCT.....AACTGGTGC
292 bp
CLN5/8 double knockout and CLN5/6/8 triple knockout edited sequence
ATAGCTGTGGACAATGAATCC
AACTGGTGC
C
WT
CLN8 KO
Relative abundance (/WT)
****
BMP 14:0_18:1
BMP 16:0_16:0
BMP 16:0_16:1
BMP 16:0_18:1
BMP 16:0_22:6
BMP 16:1_16:1
BMP 16:1_18:1
BMP 16:1_18:2
BMP 16:1_22:6
BMP 18:0_18:1
BMP 18:1_18:1
BMP 18:1_18:2
BMP 18:1_20:1
BMP 18:1_20:2
BMP 18:1_20:3
BMP 18:1_20:4
BMP 18:1_22:2
BMP 18:1_22:3
BMP 18:1_22:4
BMP 18:1_22:5
BMP 18:1_22:6
BMP 18:2_18:2
BMP 18:2_20:3
BMP 18:2_22:6
BMP 20:4_22:6
BMP 20:5_22:6
BMP 22:5_22:5
BMP 22:5_22:6
BMP 22:6_22:6
D
WT
CLN6 KO_cl1
CLN6 KO_cl2
Fold change (to 0μM)
Fold change
16:0_18:1
16:0_22:6
16:1_18:1
16:1_18:2
16:1_20:3
16:1_22:6
18:0_18:1
18:1_18:2
18:1_20:1
18:1_20:2
18:1_20:3
18:1_20:4
18:1_22:2
18:1_22:3
18:1_22:4
18:1_22:6
18:2_18:2
18:2_20:3
18:2_22:6
20:4_22:6
20:5_22:6
22:5_22:5
22:5_22:6
S,S-GPG (μM)

bioRxiv preprint doi: https://doi.org/10.64898/2026.10.07.757477; this version posted October 9, 2026. The copyright holder for this preprint (which was not certified by peer review) is the author/funder, who has granted bioRxiv a license to display the preprint in perpetuity. It is made available under a CC-BY 4.0 International license.

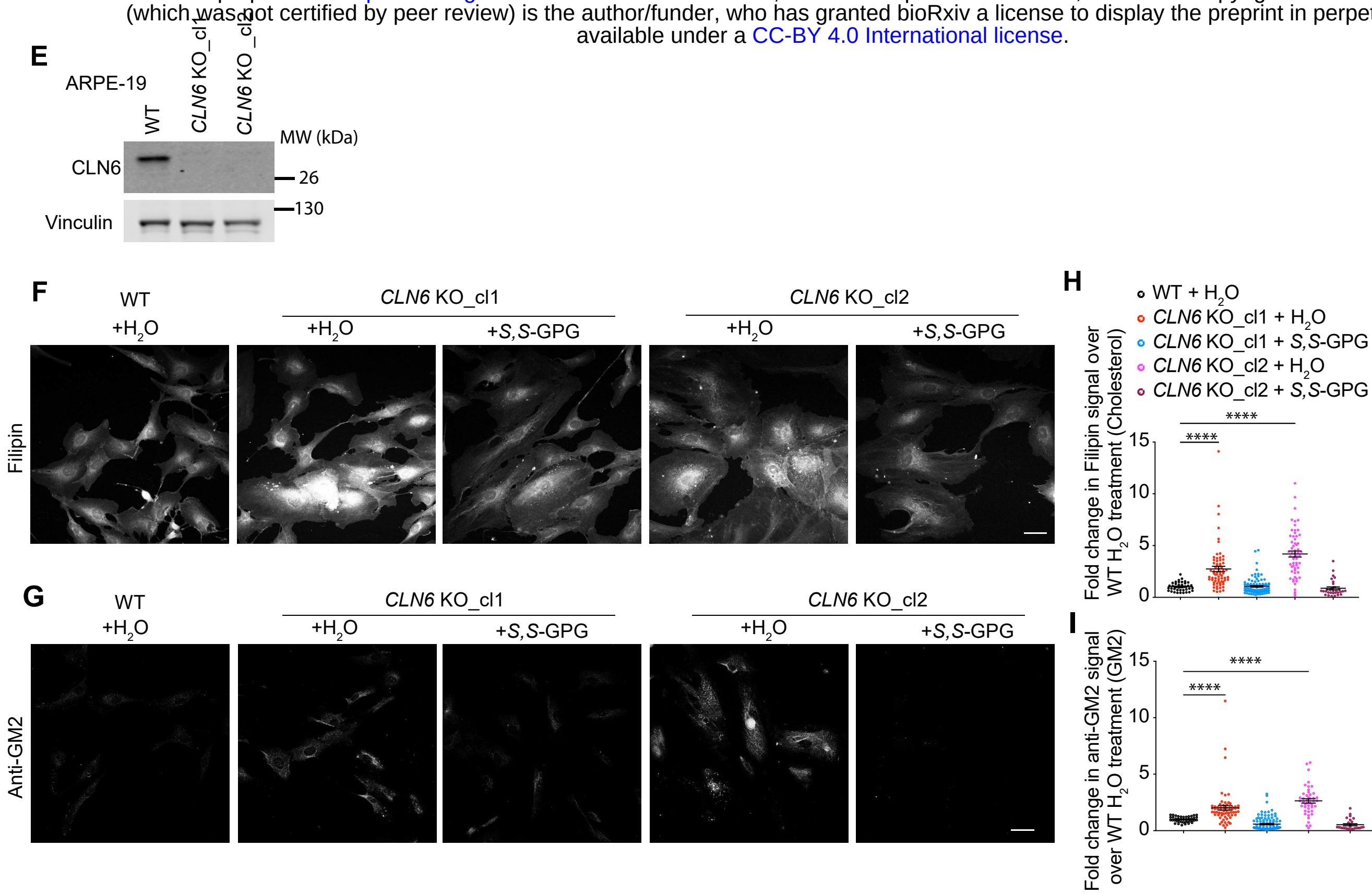

bioRxiv preprint doi: https://doi.org/10.64898/2026.10.07.757477; this version posted October 9, 2026. The copyright holder for this preprint (which was not certified by peer review) is the author/funder, who has granted bioRxiv a license to display the preprint in perpetuity. It is made available under a CC-BY 4.0 International license.

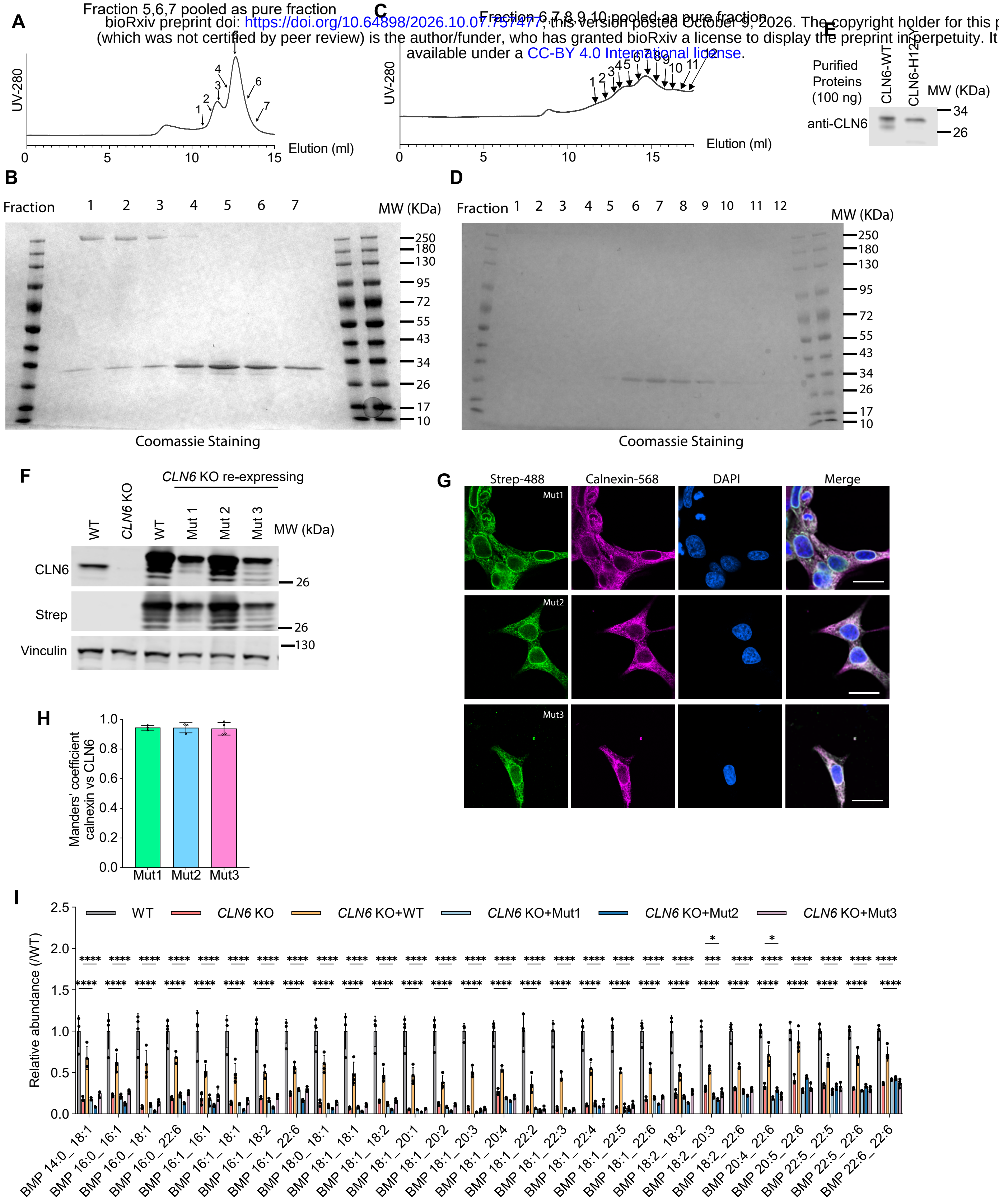

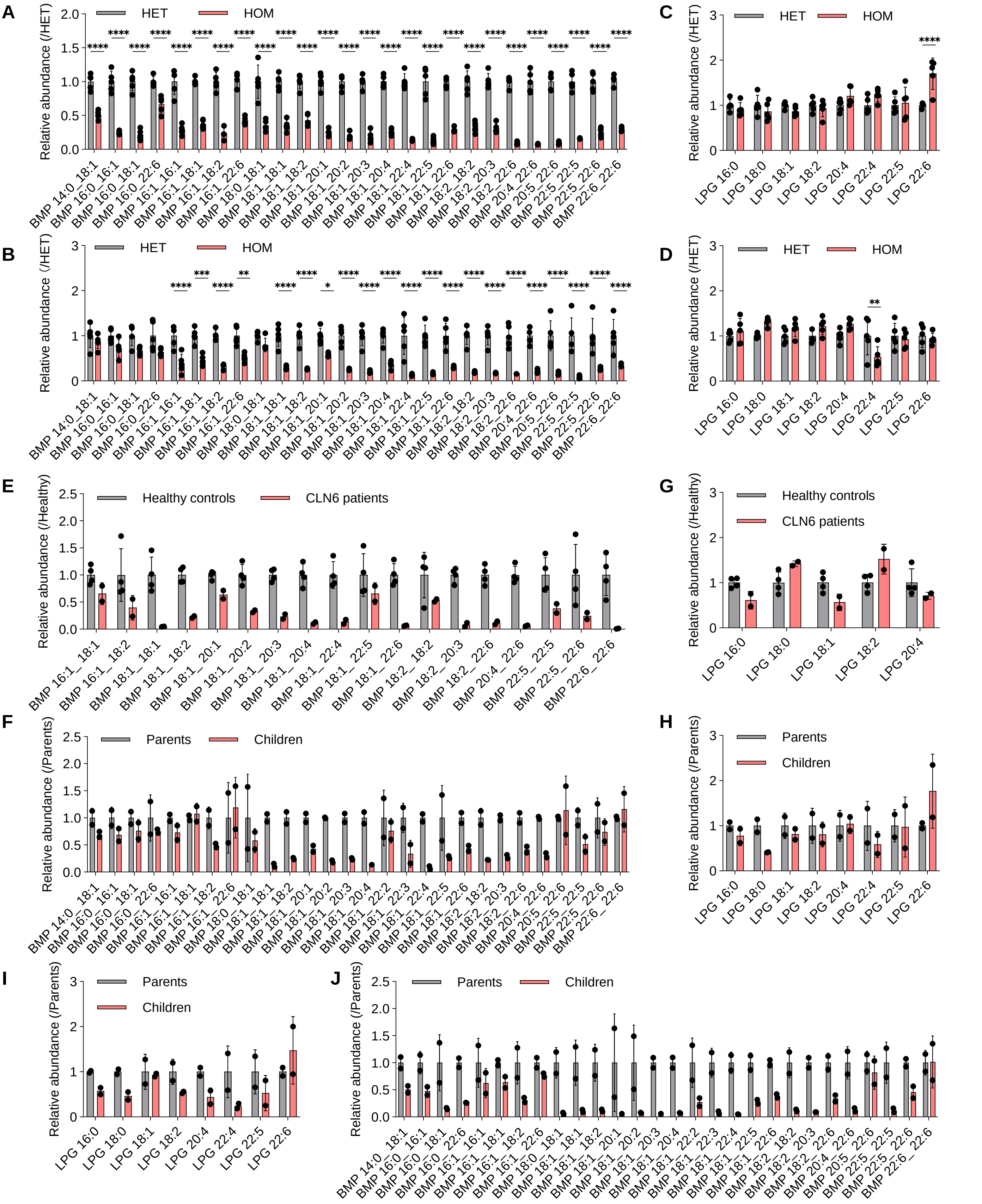

A
Relative abundance (/HET)
HET
HOM
****
BMP 14:0_18:1
BMP 16:0_16:1
BMP 16:0_18:1
BMP 16:0_22:6
BMP 16:1_16:1
BMP 16:1_18:1
BMP 16:1_18:2
BMP 16:1_22:6
BMP 18:0_18:1
BMP 18:1_18:1
BMP 18:1_18:2
BMP 18:1_20:1
BMP 18:1_20:2
BMP 18:1_20:3
BMP 18:1_20:4
BMP 18:1_22:4
BMP 18:1_22:5
BMP 18:1_22:6
BMP 18:2_18:2
BMP 18:2_20:3
BMP 18:2_22:6
BMP 20:4_22:6
BMP 20:5_22:6
BMP 22:5_22:5
BMP 22:5_22:6
BMP 22:6_22:6
B
Relative abundance (/HET)
HET
HOM
****
***
**
*
C
Relative abundance (/HET)
HET
HOM
****
LPG 16:0
LPG 18:0
LPG 18:1
LPG 18:2
LPG 20:4
LPG 22:4
LPG 22:5
LPG 22:6
D
Relative abundance (/HET)
HET
HOM
**
E
Relative abundance (/Healthy)
Healthy controls
CLN6 patients
BMP 16:1_18:1
BMP 16:1_18:2
BMP 18:1_18:1
BMP 18:1_18:2
BMP 18:1_20:1
BMP 18:1_20:2
BMP 18:1_20:3
BMP 18:1_20:4
BMP 18:1_22:4
BMP 18:1_22:5
BMP 18:1_22:6
BMP 18:2_18:2
BMP 18:2_20:3
BMP 18:2_22:6
BMP 20:4_22:6
BMP 22:5_22:5
BMP 22:5_22:6
BMP 22:6_22:6
G
Relative abundance (/Healthy)
Healthy controls
CLN6 patients
LPG 16:0
LPG 18:0
LPG 18:1
LPG 18:2
LPG 20:4
F
Relative abundance (/Parents)
Parents
Children
BMP 18:1_22:2
BMP 18:1_22:3
H
Relative abundance (/Parents)
Parents
Children
I
Relative abundance (/Parents)
Parents
Children
J
Relative abundance (/Parents)
Parents
Children
